# Research on Anti-Slip and Wear Resistance of Concrete Pavement After Optimization of Stone-Planting Process

**DOI:** 10.3390/ma18102210

**Published:** 2025-05-10

**Authors:** Jianbo Yuan, Zezhong Feng, Pei Cui

**Affiliations:** School of Traffic and Transportation Engineering, Changsha University of Science & Technology, Changsha 410114, China; yuanjb01@163.com (J.Y.); 22101030102@stu.csust.edu.cn (P.C.)

**Keywords:** road construction, cement concrete, anti-slip properties, friction coefficient, depth of construction

## Abstract

This research was conducted to improve the slip and abrasion resistance of concrete pavements by introducing the stone-planting process into concrete pavements. The anti-slip and abrasion resistance of stone-planted concrete pavements was investigated by studying the type of stone, particle size, stone spreading rate, stone indentation depth, and concrete surface treatment process parameters in the stone-planting process. The slip resistance of stone-planted concrete pavements was investigated using a pendulum tribometer and hand laying sand test. The fort Kentucky fretting abrasion test was used to study the abrasion resistance of stone-planted concrete pavement. The resulting surface had the following characteristics: the stone material was dolerite, the stone granularity was 13.2 mm, the stone spreading rate was 40%, the indentation depth was 0.8, and the surface of the stone-planted concrete pavement had better anti-slip and abrasion resistance. Therefore, the stone-planting process can effectively improve the anti-slip and wear-resistant performance of concrete pavement, improve the service life of pavement, and provide a scientific basis for research into long-lasting surface pavement.

## 1. Introduction

With the rapid development of the transportation industry, the safety of driving has received widespread attention [1]. In particular, the hard material on the surface of the cement concrete pavement of highway tunnels, under frequent vehicle loading [2], gradually wears out, resulting in a rapid decline of its anti-skid performance, which seriously affects the safety of driving [3,4]. As tunnels are in a dark state for a long time, a layer of water film inevitably exists on the tunnel road’s surface. If the interior of the tunnel consists of asphalt pavement, which is prone to water damage, it will suffer from accelerated asphalt aging and a reduced service life [5]. Tunnel entrance and exit sections are traffic-accident-prone areas due to the frequent acceleration or deceleration of vehicles, resulting in rapid wear and tear of the road surface. Considering the high risk of traffic accidents in tunnels, ensuring good adhesion between the wheels and the road surface, as well as shortening the braking distance of vehicles, is crucial for the safety of driving in tunnels [6]. Therefore, cement concrete pavements are mostly used in tunnels, and the use of high-flow concrete can improve safety performance in tunnels. Due to the enclosed environment of road tunnels, water from vehicles and tunnel arches does not evaporate easily, keeping the pavement wet for long periods and making anti-slip properties crucial for cement concrete’s road performance [7,8]. In humid environments, the surface of cement mortar crystallizes easily, forming a smooth surface, which will lead to the phenomenon of a “mirror” on the road’s surface. In addition, contaminants on the pavement tend to cover the pavement surface, which in turn affects the coefficient of friction between tires and pavement [9,10]. Therefore, it is important for engineers and researchers to understand and improve the skid resistance of cement concrete pavements in road tunnels.

Due to its own high-flow characteristics, high-fluidity concrete can solve the problems caused by the ease of construction of ordinary concrete, thus reducing construction cost and construction difficulty [11,12]. Meanwhile, high-flow concrete consumes a large amount of other solid waste such as fly ash in its production and preparation. This promotes the production of low-carbon concrete using a large amount of different solid wastes including recycled aggregates from waste concrete, plastics and rubber, tailings and tunnel slag, and biochar from different organic wastes [13]. High-fluidity concrete has had decades of development around the world, and a series of studies have been conducted by scholars at home and abroad around high-fluidity concrete [14]. For example, Zhao et al. [15] made high-flow concrete by using solid waste mixed with fly ash, thus realizing the reuse of waste. Zhang et al. [16] studied high-flow concrete mixed with steel fiber from macroscopic and microscopic perspectives, and it was found that fly ash and silica fume could reduce the void of the concrete matrix to a different extent, thus strengthening the bond between steel fibers and the concrete matrix and improving the mechanical properties of the high-flow concrete. Meanwhile, a study by Choi et al. [17] showed that by mixing superplasticizers and air-entraining agents with a reduced water-to-cement ratio, concrete containing 50% fly ash had high flowability and guaranteed early strength, reaching the target strength at a curing age of 28 days. Other studies [18,19] have shown that the compressive strength of concrete containing 40–60% ground fly ash increased with curing time, and the tensile strength was lower at 28 days curing time but reached the target at 56 days of curing. Hence, there is a need to select high-flow concrete for paving roads.

Pavement graveling is a new technique for improving the surface texture of concrete pavements. This technique requires the spreading of artificial stone-planting material on the surface of concrete pavement when the construction is initially completed, and then the stone-planting material is pressed into different depths to change the texture and shape of the pavement, so as to achieve the requirement of improving the anti-skid performance of the pavement. Based on this, Feng et al. [20] tested the mechanical properties by adding mudstone to cement, and the results showed that the California bearing ratio of the added mudstone was more than 20% higher than that of the unadded cement specimen. The stone-planting process is a concrete surface texture improvement technique, and the current research on its application in roads is still not deep enough.

In order to assess the slip resistance of cement pavement, it is usually necessary to conduct relevant tests in a laboratory to evaluate the performance of the pavement by simulating the friction performance, slip resistance, and other indicators of different pavement materials [21]. These tests can provide a scientific basis for pavement design and construction and guarantee the safety and stability of roads [22]. At present, fixed-point measurement and continuous measurement are the main methods for measuring the coefficient of friction of pavement [23,24]. The fixed-point measurement mainly includes the British Pendulum Tester (BPT) and Dynamic Friction Tester (DFT). There are four types of continuous measurements based on the slip rate setting mode: lateral force measurements, hold-up wheel measurements, fixed slip rate measurements, and variable slip rate measurements. The BPT is a dynamic pendulum impact type designed to measure the energy loss of a rubberized slider that is propelled on the pavement. The measured value is the British Pendulum Number (BPN), an indication of the friction performance of a tire interacting with the road surface, which is obtained using equipment and procedures standardized by ASTM E 303-93 in the field or in the laboratory [25]. The BPT is very portable and easy to operate. The test procedure requires on-site traffic control and lane closures. BPN reflects the skid resistance of the pavement at low speeds. However, the skid resistance of pavement is directly related to the safety and comfort of driving. According to the newly released JTG F80/l-2017 Standard for Quality Inspection and Evaluation of Highway Engineering, the corresponding indexes are required [26]. Pavement skid resistance has become one of the main acceptance items in road design specifications, construction process specifications, and maintenance regulations. The U.S. Department of Transportation in Virginia evaluates the skid resistance of pavements by using the laser texture tester and sand spreading methods [27]. The California Department of Transportation in the United States utilizes a laser ranging vehicle to measure the constructional depth of pavements [28]. In the future, with the development of high-speed transportation, roads must have good skid resistance.

To address the above issues, this paper will carry out a study on the skid resistance and abrasion resistance of high-fluid concrete stone-planted pavements. The pendulum friction coefficient and hand sanding method are utilized to establish the correlation between high-flow concrete and the stone-planting process. The fort Kentucky flyaway abrasion test was utilized to study the abrasion resistance of concrete pavements. The slip and abrasion resistance of high-flow concrete pavements was studied in terms of type of stone-planting material, particle size, spreading rate, and depth of construction, in order to improve the service life of pavements and contribute to the research on concrete pavement slip and abrasion resistance.

## 2. Materials and Tests

### 2.1. Materials

#### 2.1.1. Cement

The cement used in this paper is from Southern Cement Co., Ltd., UK and meets the Technical Rules for Highway Cement Concrete Pavement Construction (JTG/T F30-2014) [29]. In accordance with the Test Procedure for Cement and Cement Coagulation in Highway Engineering (JTG E30-2005) [30], the main indicators of cement were measured, and the results are shown in Table 1.

#### 2.1.2. Silica Fume

Silica fume is a dust recovered from the smelting of other alloys such as silicon metal. The apparent density of silica fume is measured as 2400 kg/cm^3^, which meets the requirements of GB/T27690-2023 [31] Silica Fume for Mortar and Concrete.

Silica fume can fill the pores between cement particles, and at the same time, the hydration product generates gel, and the alkaline material magnesium oxide generates gel. In addition, due to the cement particles being filled with silica fume particles, silica fume particles can replace the water in the cement mortar voids, and these replacements become free water. Silica fume improves concrete fluidity by filling voids and releasing free water, enhancing mix workability.

#### 2.1.3. Coarse Aggregates

The coarse aggregate gravels selected for the test were 5 mm~1.0 mm gradation and 1.0 mm~2.0 mm gradation, respectively, and the basic physical properties measured after mixing the two types of gravels together are shown in Table 2.

#### 2.1.4. Fine Aggregates

River sand was used in the test, and the physical index test results are shown in Table 3.

Sieve analysis revealed that the particle size distribution of river sand falls within the range of 9–14 mm. Based on the calculated fineness modulus (Mx = 2.9), which lies within the range of 2.3–3.0, river sand is classified as medium sand.

#### 2.1.5. External Admixtures

Naphthalene, a highly efficient water reducing agent selected for external admixture, was provided by Shanghai Chenqi Chemical Technology Co. (Shanghai, China). The test report is shown in Table 4.

#### 2.1.6. Stone Planting

Three different types of crushed stone (limestone, gabbro, and basalt) were mainly selected for the stone-planting material, and the results shown in Table 5 were obtained through physical property tests.

### 2.2. Stone-Planting Process

In this study, the indoor molding method was used, the mixing method was manual mixing, and the size of the mill used was 40 × 40 × 5 cm. The specific operation is shown below.

**Figure 1:**
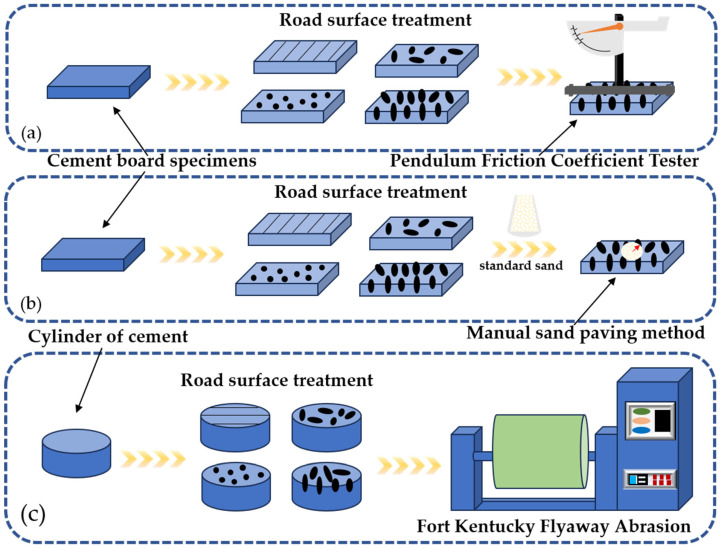
Research route on anti-skid and wear resistance of stone-planted concrete pavement: (**a**) Pendulum Friction Coefficient Test, (**b**) Manual Sand-Paving Method, (**c**) Fort Kentucky Flyaway Abrasion Test.

(1) After assembling the abrasives, brush the appropriate amount of release agent around the inner wall of the mold to prevent the concrete time from adsorbing to the abrasives or the ground when hardening and demolding. (2) Pour the coarse and fine aggregates and cement into the mixing board in turn, mix with iron rowan until homogeneous, add the external admixture, pour in the appropriate amount of water to mix slowly, wait until the water is absorbed, pour in the rest of the water, and continue to mix evenly, mixing time of about 4–5 min. (3) Put the mixed concrete into the pounding rod and pound it from the edge to the center along the spiral line about 25 times. Use the tamping rod to gently tap around the specimen to make the specimen vibrate densely, and at the end of loading, use the scraper to scrape away the excess mix and smooth the surface of the specimen. (4) Adopt the spreading area of the stone-planting material to control the amount of stone spreading, use the area coverage to equalize the density of spreading, and use the mass of spreading stone material to determine the spreading area of stone-planting material. (5) Using absorbent paper, check the surface of the test piece slurry or wait until the test piece is stable and water secretion no longer occurs, and then the stone-planting material should be evenly spread on the surface of the test piece. (6) After uniformly spreading the test piece on a flat iron plate, measure the height (H) at three points using a straightedge and take the average. Subtract 5 cm from H to determine the planting material’s height on the test piece surface. Then, subtract this height from the weighed planting material size and compare it to the planting material grading (i.e., the indentation depth). Based on the required depth for each specimen group, slowly adjust the height until the compression depth meets the requirements. Finally, remove the iron plate. (7) The specimens are numbered and placed in the conditioning room for standard maintenance. The study route is shown in Figure 1.

### 2.3. Stone-Planting Parameters

In order to understand the skid resistance and abrasion resistance performance of stone-planted concrete pavement, the stone-planted pavement was investigated by studying three parameters: stone spreading area, indentation depth, and type of stone planted.

#### 2.3.1. Stone Spreading Area

In order to investigate the effect of stone particle size on the stone-planted concrete pavement, two kinds of particle sizes, 13.2 mm and 9.5 mm, were designed for the configuration of single grading and composite grading. Different ratios of 9.5 mm single-size crushed stone and 13.2 mm single-size crushed stone were used, 9.5 mm/13.2 mm = 1:1 (9.5 and 13.2 for each half of the composite size graded crushed stone) and 9.5 mm/13.2 mm = 2:1 (9.5 and 13.2 quality ratio of 2:1 composite size graded crushed stone) to produce four kinds of stone grain size. Because the size of the stone material is not uniform, this paper controls of the stone material spreading amount to control the withdrawal of stone material area coverage, and the area coverage is equal to the withdrawal of density, with the spreading of the mass of the stone material being used to determine the spreading area of the stone-planted material. The density of the stone material spreading test results is shown in Table 6. The ratio of the area occupied by the stone-planting material to the total area of the stone-planting pavement, in %, is one of the main indicators for evaluating the surface structure of stone-planting concrete pavement.

#### 2.3.2. Indentation Depth

**Figure 2:**
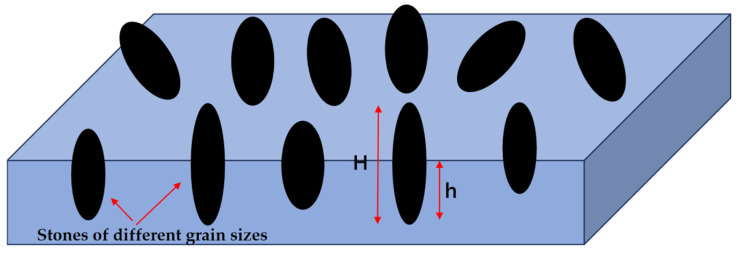
Schematic diagram of stone-planted cement concrete pavement structure.

Depth of indentation: h/H, dimensionless unit, as shown in Figure 2, and the ratio of the length (h) of the part pressed into the cement concrete pavement to the particle size (H) of the planting material. A larger value for the depth of indentation indicates that the planting material is embedded in the surface of the cement concrete pavement more deeply.

The homogeneous cement concrete was loaded into the corresponding size of the grinding tool, and the excess cement concrete was scraped off with a scraper, and then the surface of the specimen was leveled by manual vibration, the floating slurry formed on the surface of the specimen during the vibration was sucked off with absorbent paper, and the stone material of the corresponding particle size was weighed and spread evenly on the surface of the specimen. Then, the exposed size of the stone material was measured three times, and the size of the stone material was used, and the depth of the stone-planting material was subtracted from the size of its structure as h. Finally, the ratio of h and H was used to obtain the indentation depth of the stone-planted specimen.

#### 2.3.3. Types of Planting Stone

Types of planted stone material mainly include three different types of crushed stone, limestone, gabbro, and basalt, and each type of crushed stone produced stone-planted pavement of a different nature.

#### 2.3.4. Surface Treatment of Planted Stones

**Figure 3:**
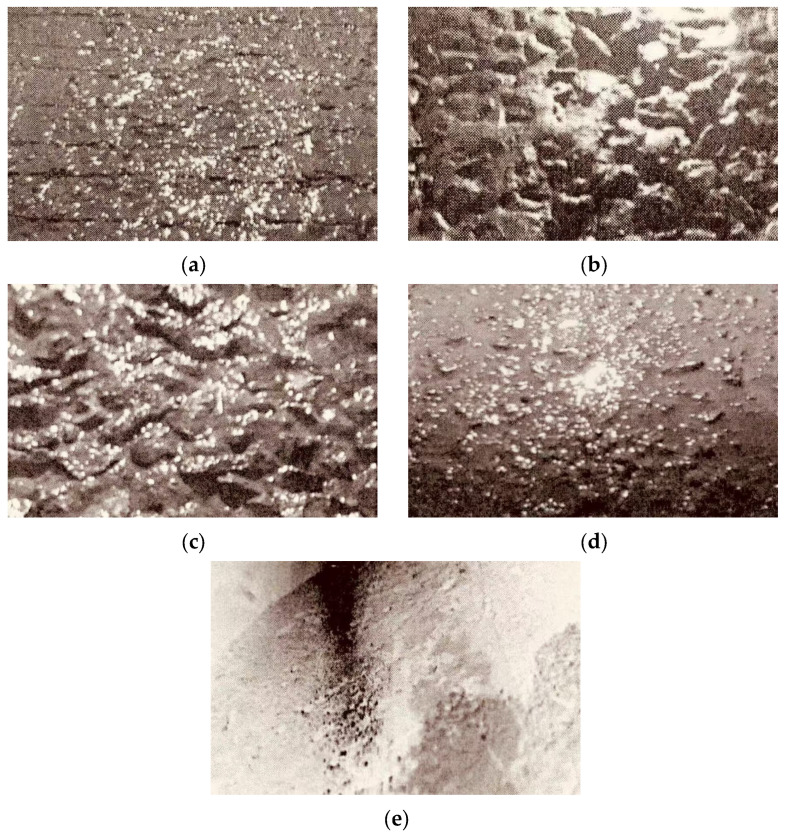
Concrete samples under the stone-planting process: (**a**) grooved pavement; (**b**) exposed stone pavement; (**c**) stone-planted pavement; (**d**) rough surface pavement; (**e**) glossy pavement.

Pavement stone planting is a new process of cement concrete surface texture treatment that requires the surface of newly constructed cement concrete to be evenly spread to meet the specific requirements of the stone-planting material, which is applied manually or mechanically by pressing the stones to a specific depth, which can significantly improve the surface texture structure. The stone-planting process samples are shown in Figure 3.

### 2.4. Planted Stone Anti-Slip and Abrasion Test

#### 2.4.1. Pendulum Friction Coefficient

The instruments used were from Jiangsu Muyang Luda Highway Instrument Factory in Jiangsu, China. Pendulum friction coefficients are obtained by determining them with a pendulum tribometer, as shown in the following procedure.

Place the instrument on the measuring point and turn the screw to center the leveling bubble. Loosen the fixing handle, turn the lifting handle so that the pendulum is raised to swing freely, and then tighten the handle. Move the pendulum to the right and press the release switch. Press the release switch to move the pendulum to the left, drive the pointer upward, reach the highest position after the fall, and catch the pendulum with the left hand; at this time, the pointer should point to zero. The distance between the two contact points of the rubber sheet and the road surface should be 126 mm; then, place the pendulum in the horizontal release position. Pour water onto the concrete pavement specimen and press the release switch so that the pendulum slides over the pavement. The pointer can indicate the value of the coefficient of friction of the pavement. Repeat the measurement three times and record the value each time.

**Figure 4:**
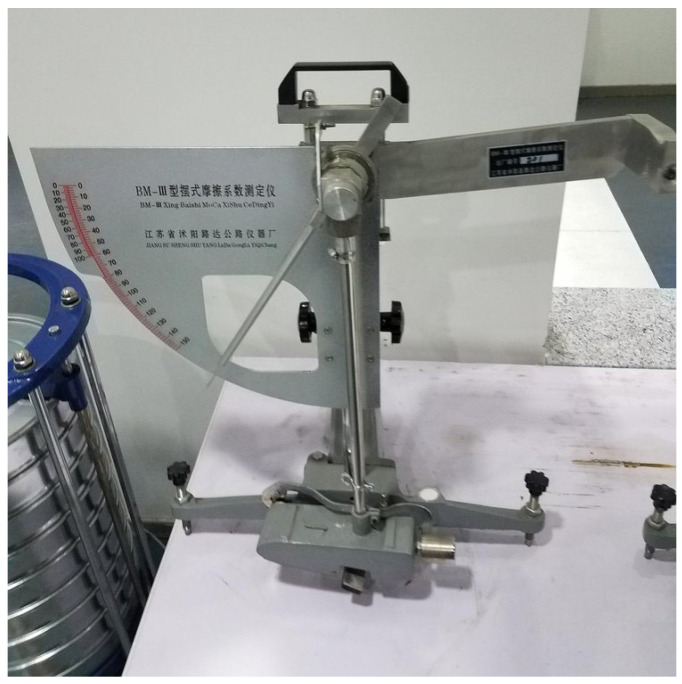
Pendulum friction coefficient tester.

Figure 4 shows the pendulum friction meter; after obtaining data through the instrument, the pendulum friction coefficient of the specimen can be obtained according to the following formula [32]:

(1) $$F=\frac{WHZ}{PDM}\times 100$$

where:

F—value of the pendulum;
W—mass of the pendulum in grams (g);
H—distance from the center of gravity of the pendulum to the axis of oscillation in millimeters (mm);
Z—the vertical distance below the zero position of the dial in millimeters (mm);
P—the positive static pressure of the rubber sheet on the surface of the object to be measured, in Nm (N);
D—sliding length of the rubber sheet on the road surface, in millimeters (mm);
M—distance from the tip of the dial pointer to the center of rotation of the pointer, in millimeters (mm).

#### 2.4.2. Manual Sand-Paving Method

The manual sand-paving method involves spreading sand on the road surface and then calculating the ratio of the sand volume filling the voids to the area covered by a standard measuring cylinder after flattening. The sand used in the cylinder is medium-grained, with a particle size distribution between 0.5 and 1 mm, complying with ASTM E965. The texture depth (TD) of the pavement can be determined using this sand-patch method. The measurement is repeated three times, with each result recorded. The pavement’s TD is typically used to evaluate road surface skid resistance, calculated using the following formula [33]:

(2) $$TD=\frac{1000V}{\pi D^{2}/4}$$

where:

TD—texture depth of concrete surface in millimeters (mm);
V—volume of sand and gravel, in millimeters (mm);
D—sand surface diameter, in centimeters (cm).

#### 2.4.3. Fort Kentucky Flyaway Abrasion Test

**Figure 5:**
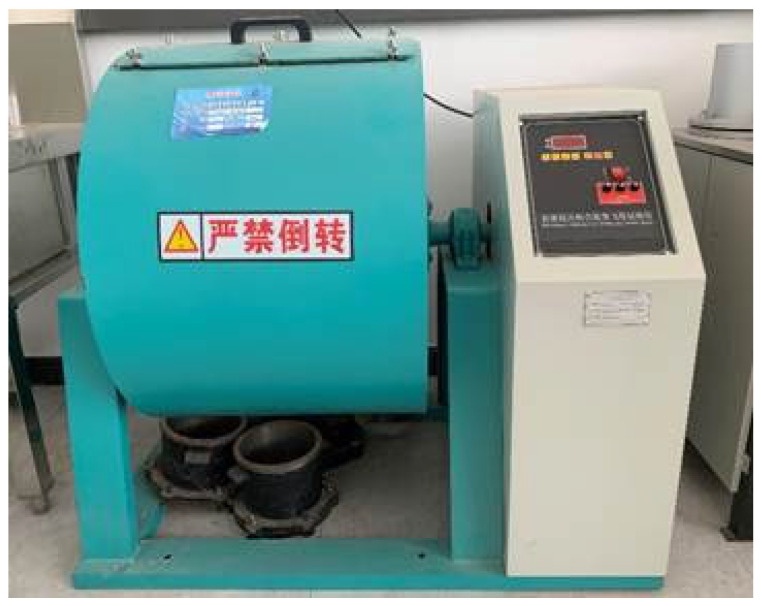
Fort Kentucky flyaway abrasion.

The fort Kentucky flyaway abrasion test is used to evaluate the asphalt mixture abrasion test under traffic loading, as shown in Figure 5. In this study, the test was conducted along the same lines, but the asphalt mixture specimen was modified to stone-planting concrete, none of the model shapes and sizes were changed, and the abrasion resistance of stone-planting concrete was studied using the fort Kentucky flying splash abrasion meter. The instrument used is from Cangzhou Taiding Hengye Experimental Instrument Co., Ltd. in Hebei Province, Cangzhou, China.

In this study, the method is based on the use of molds to directly form cylindrical specimens with a diameter of 101.6 mm and a height of 63.5 mm, which were firstly incubated in a constant temperature water bath at 20 °C for 20 h. Then, the specimens were taken out of the bath, the surface water was gently wiped off with a clean, soft towel, and the mass of each specimen was weighed. Then, the specimens were placed into the Los Angeles testing machine and rotated at 30–33 r/min for 100/300/500/1000 revolutions; finally, the specimens and their broken pieces were taken out and then weighed to determine their the residual mass. These measurements were repeated three times, and the values were recorded each time, using the formula shown below:

(3) $$∆s=\frac{m_{0}-m_{1}}{m_{0}}\times 100$$

where:

∆s—cement concrete flyaway losses (%);
m_0_—quality of the specimen before the test (g);
m_1_—mass of specimen remaining after test (g).

## 3. Results and Analysis

This paper used the sand-laying method test in order to study the type of stone material, particle size, spreading rate, compression depth, and surface treatment process on the pavement construction depth, in order to determine the change in the anti-slip performance of the pavement. The pendulum friction method was used to analyze the influence of different stone-planted material parameters on the high-flow state stone-planted pavement’s skid resistance. The influence of different stone-planting material parameters on the abrasion resistance of high-fluid stone-planting pavement was analyzed through the Kentaburg flyaway abrasion test.

### 3.1. Analysis of Slip Resistance of Stone-Planted Cement Concrete

#### 3.1.1. Effect of Stone Type on Anti-Slip Properties

**Figure 6:**
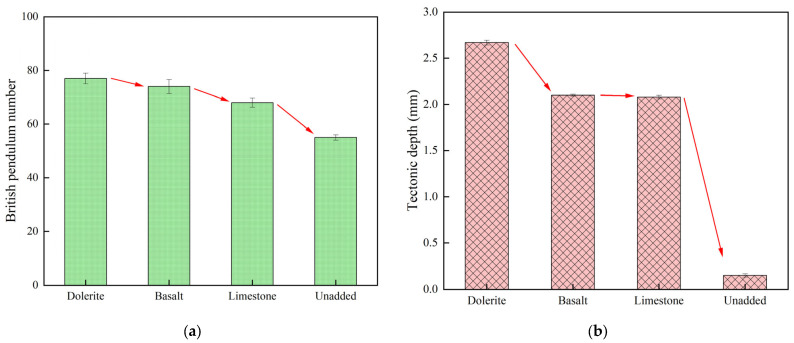
Stone type with pendulum value and texture depth test results: (**a**) Pendulum value, (**b**) Tectonic depth.

The effect of different stone materials on the anti-skid performance of cement pavement was investigated by selecting dolerite, basalt, and limestone for cement pavement stone-planting treatment. The results of pendulum and TD values of cement concrete specimens with different types of stone planting are shown in Figure 6.

As shown in Figure 6, the selection of dolerite may lead to a slight increase in the constructional depth and pendulum value of concrete specimens under the same grading conditions. The pendulum value of the concrete specimens without stone-planting treatment was 55, and due to the addition of dolerite, the pendulum value was increased to 77, and the slip resistance also increased. The slip resistance increased by 40% compared to the untreated concrete specimens. The concrete specimens without stone-planting treatment had a texture depth of 0.15, while the texture depth of the specimens with the addition of dolerite was as large as 2.67, which is a significant increase in the texture depth. This may be due to the fact that the distribution of larger stones in concrete leads to changes in the concrete structure, which affects the skid resistance of the concrete pavement. This finding can provide an important reference for the design and construction of concrete pavements, especially in the selection of phyllite materials, and the effect of the particle size of the stone on the pavement’s performance needs to be considered.

#### 3.1.2. Effect of Stone Particle Size on Anti-Slip Properties

**Figure 7:**
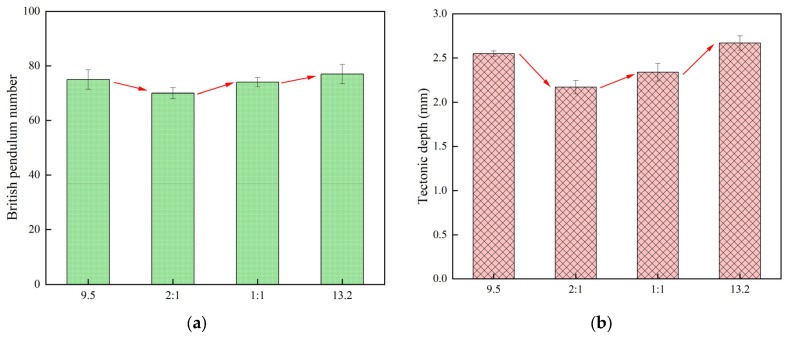
Stone grain size and pendulum value and texture depth test results: (**a**) Pendulum value, (**b**) Tectonic depth.

The effect of different grain sizes on the anti-slip properties of cement pavement was investigated by selecting dolerite in sizes of 9.5 mm and 13.2 mm. At the same time, the effect of particle size admixture on the slip resistance of cement pavement was investigated at different ratios, including 9.5 mm/13.2 mm = 1:1 and 9.5 mm/13.2 mm = 2:1. The results of the pendulum and TD values of cement concrete specimens with different stone particle sizes are shown in Figure 7.

From Figure 7, it can be seen that selecting a single stone grading of 13.2 mm can improve the slip resistance of high-flow concrete. However, under the 9.5 mm and 13.2 mm composite grain size conditions, the surface flatness is difficult to ensure, resulting in smaller construction depth and pendulum values. This difference may be due to the different energy dissipation by the pendulum tribometer under different grain size conditions. The pendulum values in the single grain size condition are large relative to the pendulum values in the composite grain size condition. A single stone size of 13.2 mm increased the pendulum value by 10% for the composite size. These findings help withy understanding the effect of stone gradation on concrete properties. Therefore, selection of stone gradation with a single grain size of 13.2 mm is beneficial for improving the slip resistance of high-flow concrete.

#### 3.1.3. Effect of Stone Cover on Anti-Slip Performance

**Figure 8:**
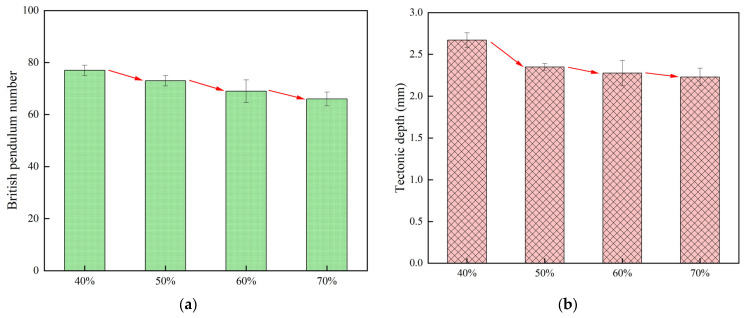
Stone spreading area with pendulum value and construction depth test results: (**a**) Pendulum value, (**b**) Tectonic depth.

The concrete specimens were planted with dolerite using different coverage ratios. The anti-slip performance of the stone-planted concrete specimens with different cover areas was tested by using the pendulum friction coefficient meter and hand sand-paving test, and the test results are shown in Figure 8.

As can be seen from Figure 8, at a smaller spreading rate, there are more voids between stones and more sand to be filled, so the construction depth and pendulum value will be relatively larger. At a larger spreading rate, there are fewer voids between stones, and the specimen surface is flatter. Therefore, the texture depth and pendulum value are relatively small. With the increase in spreading rate, the pendulum value of the specimen gradually decreases, up to 14.3%. The increase in the spreading rate from 40% to 70% decreased the pendulum value of the specimen by 14.3%, indicating that an increase in the spreading rate of the specimen will show a decrease in the slip resistance of the specimen.

#### 3.1.4. Effect of Stone Indentation Depth on Anti-Slip Performance

**Figure 9:**
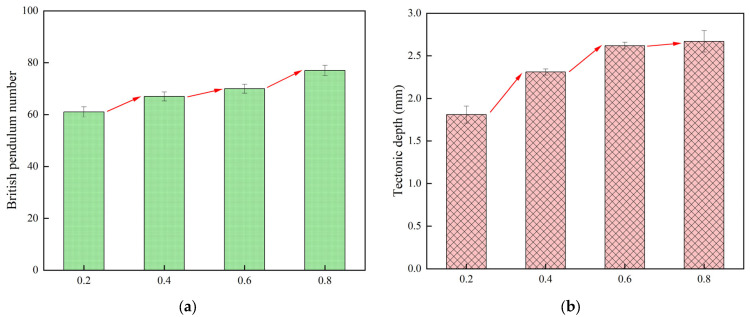
Stone indentation depth with pendulum value and texture depth test results: (**a**) Pendulum value, (**b**) Tectonic depth.

The anti-skid performance was investigated by different depths of stone indentation in the specimens, so as to better understand the effect of stone indentation depth on the anti-skid performance of cement pavement, and the specific results are shown in Figure 9.

As shown in Figure 9, the construction depth and the pendulum value increase gradually with the increase in the indentation depth. The maximum constructional depth of 2.97 mm and the maximum pendulum value of 77 were obtained when the indentation depth was 0.8. However, when the indentation depth was 0.2, the constructional depth of 1.81 and the minimum pendulum value of 61 were obtained, and it can be found that the difference between the maximum and minimum values is relatively obvious. This is due to the fact that the smaller the depth of stone indentation, the more volume of stone-planted material is exposed to the outside, but these stone-planted materials can form excellent flatness. Therefore, as the depth of indentation increases, the depth of construction increases by 47.5% and the pendulum value increases by 26.2%.

#### 3.1.5. Effect of Surface Treatment on Anti-Slip Properties

**Figure 10:**
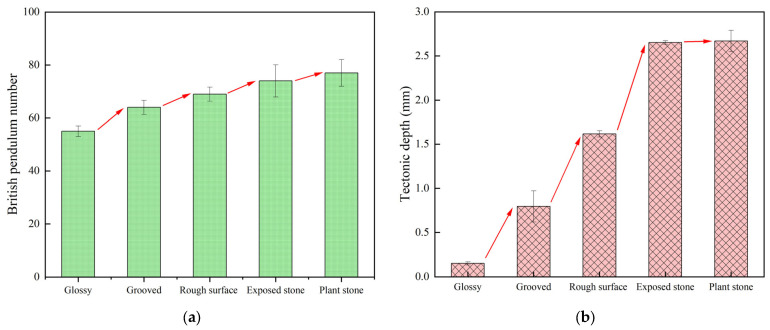
Test results of pendulum value and construction depth under different surface treatment processes: (**a**) Pendulum value, (**b**) Tectonic depth.

Five surface treatment processes, such as stone planting, stone exposure, grooving, rough surfacing, and glossing, were used to analyze their effects on the anti-skid performance of cement pavement. The tests of pavement texture depth and pendulum value under different surface treatment processes are shown in Figure 10.

By analyzing the data in Figure 10, it can be seen that the anti-slip properties of the pavement have been improved to different degrees after different surface treatment processes. Processes like rough surfacing, grooving, and stone exposure enhance both the micro- and macrostructure of road surfaces, improving the anti-skid properties of cement pavement. The anti-skid ability of the road directly affects the safety and comfort of vehicle traveling, because it can ensure that the vehicle has sufficient adhesion during traveling and reduces the braking distance, thus significantly reducing the accident rate.

According to the test results, the constructional depth and pendulum values are higher than those of the conventional glossy concrete pavement on both stone-planted concrete pavement and exposed stone concrete pavement. Planted concrete pavements and exposed stone concrete pavements have good surface structure, especially when good stone-planting material is selected, as the stone-planted structure can be maintained for a long period of time without dislodging, thus maintaining good anti-skid performance. This is due to the fact that the stone material of the stone-planted pavement is exposed on the road surface, and the main abrasion is the abrasion of the stone material.

In summary, the magnitude of anti-slip properties of different surface treatment processes is ranked as follows: stone-planted concrete pavement > exposed stone concrete pavement > grooved concrete pavement > brushed concrete pavement > glossy concrete pavement. These results provide a reference for selecting a suitable surface treatment process to enhance the anti-skid properties of the roadway, thus enhancing traffic safety and comfort.

### 3.2. Analysis of the Abrasion Resistance of Stone-Planted Cement Concrete

#### 3.2.1. The Effect of Material Type on Abrasion Performance

**Figure 11:**
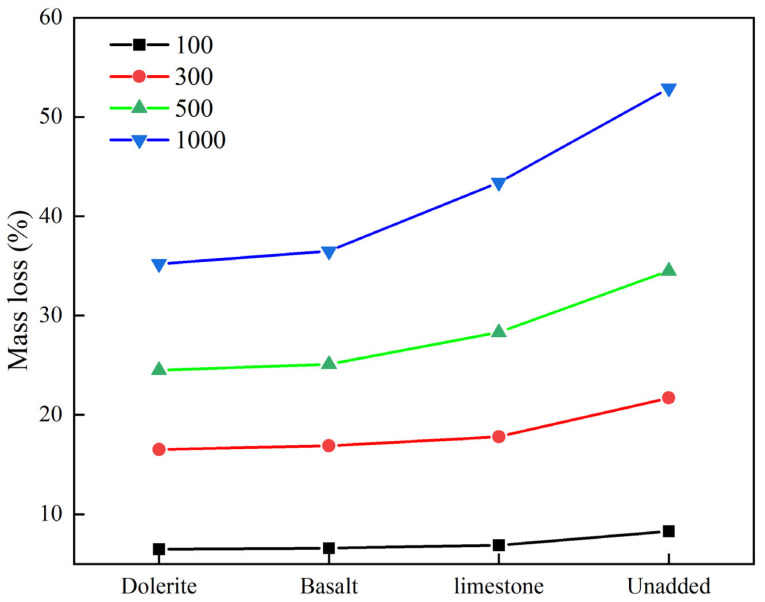
Test results of stone type and abrasion loss.

In order to study the effect of stone types on the abrasion resistance of stone-planted pavements, different stone types were investigated in the abrasion resistance of stone-planted pavements. The test results of abrasion loss of stone-planted cement concrete under different stone type conditions are shown in Figure 11.

It can be seen from the analysis of the material abrasion tests in Figure 11 that there are significant differences in the anti-abrasion properties of the different stone types. Dolerite shows the most excellent anti-abrasion characteristics, with an abrasion rate of only 35.2% under the condition of 1000 rotations, which is 17.7 percentage points lower than that of the worst-performing glossy stone material (52.9%), which is a clear advantage. Basalt followed with an abrasion rate of 36.5%, with a gap of only 1.3% with dolerite, while limestone (43.4%) and glossy stone material were in the third and fourth tiers, respectively. It is worth noting that the difference in the abrasion rate of each type of stone is relatively small (6.5–8.3%) in the case of a low number of laps, but with the increase in the number of laps, the gap in performance is gradually widened, and the extreme difference is already up to 17.7% at 1000 laps. From the engineering application point of view, for high-wear environments such as road paving, priority should be given to the use of gabbro or basalt; limestone is suitable for medium-wear requirement scenarios; and glossy stone material is only recommended for decorative and other low-wear occasions due to the poor anti-abrasion performance. It is suggested that other factors such as material cost, processing difficulty, supply stability, etc., should also be taken into account in the actual selection of materials in order to make the optimal choice.

#### 3.2.2. Effect of Stone Particle Size on Wear Resistance

**Figure 12:**
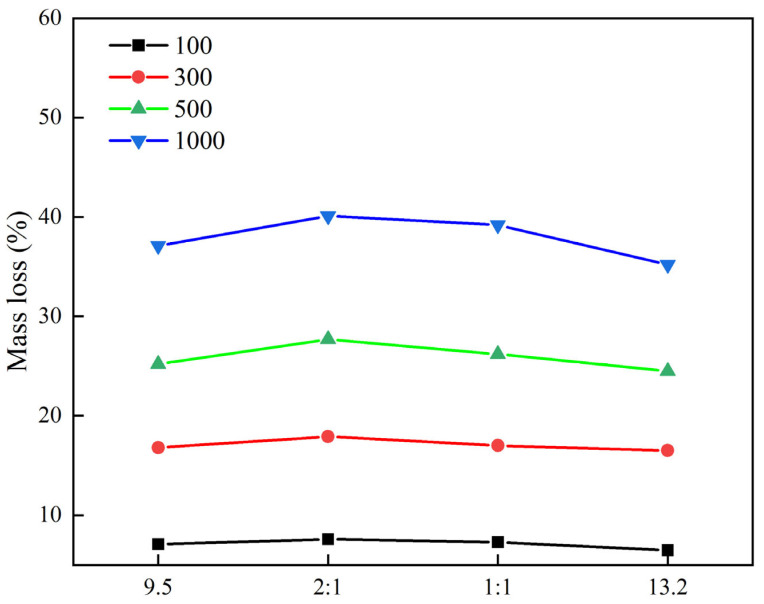
Stone grain size and abrasion loss test results.

In order to analyze the influence of stone particle size on the wear resistance of planted pavement, this experiment selected diabase for testing. Under the premise of not changing the number of raw materials and preparation process, planted concrete specimens were made, and the wear test was carried out with a Los Angeles abrasion machine. The test results are shown in Figure 12.

As shown in Figure 12, from left to right, the abrasion mass loss of the specimens with different stone grain sizes increased and then decreased after the fort Kentucky flyaway test. The 9.5 mm and 13.2 mm specimens had mass losses that were smaller than the remaining two, with the specimen with a grain size of 13.2 mm having the smallest mass loss. The particle size of the stones affects the overall mix size, because the different sizes of stones spread onto the surface of cement concrete will affect the effective contact area between the surface of the planted cement concrete road and the cement mortar. The 9.5 mm and 13.2 mm stones have a larger effective area, so that they can bond with the cement mortar, but the grading of 9.5 mm stones is smaller than the grading of 13.2 mm stones, and under the same conditions, the smaller stones have a larger effective area. Under the same conditions, the smaller size of stones has a larger effective surface area in contact with the cement paste, while the stones in contact with a vehicle can uniformly disperse the tires’ applied stress, which is closer to the ideal mode of uniform dispersion of the central mass network in the cementitious medium, and the planted stone and cement concrete interface bonding is better, so the 13.2 mm size among the four particle sizes has the smallest wear loss. Composite particle size 2:1 and composite particle size 1:1 both have 9.5 mm and 13.2 mm sizes of gravel, which makes it easy to form a similar skeleton structure, so that its effective area is smaller than the separate 9.5 mm and 13.2 mm particle sizes of the stone-planted concrete pavement.

#### 3.2.3. Effect of Stone Spreading Area on Wear Resistance

**Figure 13:**
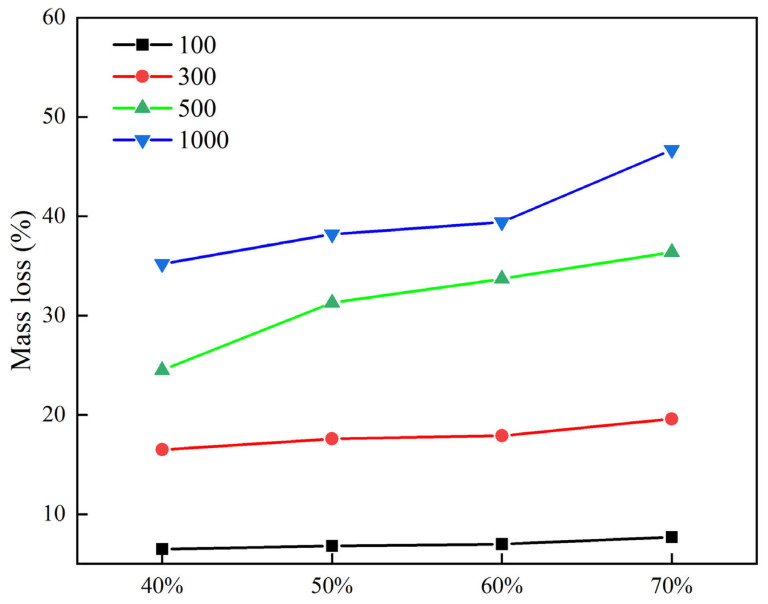
Stone spreading area and abrasion loss test results.

In order to study the effect of the stone spreading area on the abrasion resistance of stone-planted pavement, different stone spreading areas in the abrasion resistance of stone-planted pavement were investigated. The test results of abrasion loss of stone-planted cement concrete under different stone spreading area conditions are shown in Figure 13.

As shown in Figure 13, the abrasion loss of the stone-planted concrete pavement further increases with the increase in stone spreading area. When the stone type, grading, and indentation depth are certain, each stone is subjected to approximately the same stress, and as the spreading area increases, the effective area of the planted concrete pavement increases, and the pressure on the road surface further increases. When the stone and concrete bond reaches its limit, the stone-planting material will become loose in the road surface and slowly begin to fall apart. As a result, the wear loss will increase, so when the stone spreading area rate is 40% for a longer time, the specimen’s wear quality loss is the smallest, and when the spreading area rate is 70% for a longer time, the specimen’s wear quality loss is the largest.

#### 3.2.4. Effect of Stone Loading Depth on Wear Resistance

**Figure 14:**
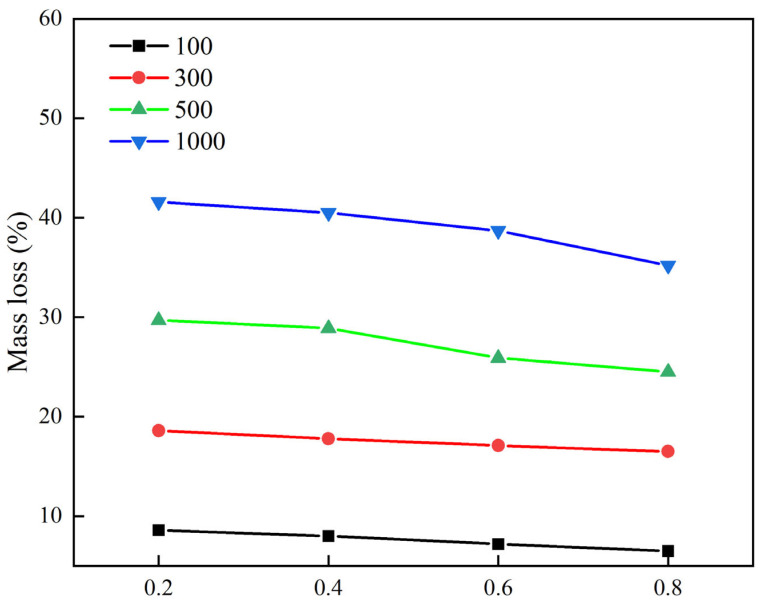
Press-in depth and wear loss test results.

In order to study the influence of stone loading depth on the wear resistance of stone-planted pavement, the wear resistance of different pressure depths is studied. The test results of stone-planted cement concrete wear loss under different stone pressure depth conditions are shown in Figure 14.

It is shown in Figure 14 that the properties of skid resistance are not identical for the same stone size for different stone indentation depths. The abrasive mass loss of the stone-planted cement concrete decreases further with the increase in the indentation depth. Under the same conditions of planting material type, stone size, and spreading area, the depth of indentation directly affects the embedding depth of the planting material. As the depth of stone embedment increases, the less exposed portion of the planting material on the outside, which can increase the contact area between the cement mortar and the planting material, increases the cementation between the planting material and the cement mortar, and there will be more cement mortar around to protect the planting material, thus effectively preventing the dislodgement of the planting material in the KENTABURG flying scatter test. Therefore, when the maximum depth of indentation is 0.8, the abrasion mass loss of the test specimen is the minimum, and when the depth of indentation is 0.2, the abrasion mass loss of the test specimen is the maximum.

#### 3.2.5. Effect of Different Surface Treatment Processes on Wear Resistance

**Figure 15:**
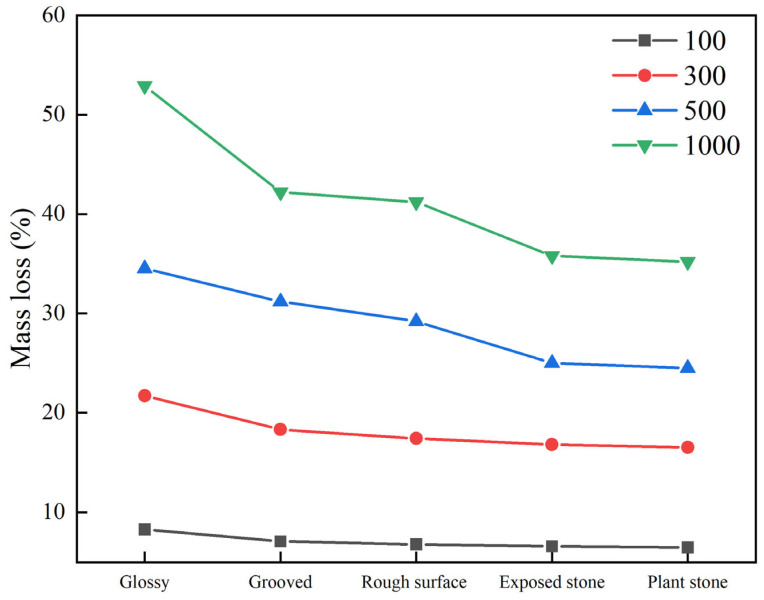
Test results of different surface treatment processes and abrasion loss.

In order to study the effect of different surface treatment processes on the abrasion resistance of stone-planted pavements, the surface treatment processes of stone in the abrasion resistance of stone-planted pavements were investigated. The test results of abrasion loss of stone-planted cement concrete under the conditions of different surface treatment processes are shown in Figure 15.

Based on the experimental data in Figure 15, the following important conclusions can be drawn from an in-depth analysis of the anti-loss performance of the different surface treatment processes: firstly, in terms of the overall trend, the loss rate of all five surface treatment processes increases significantly with the increase in the number of rotations (from 100 to 1000), which indicates that there is an obvious positive correlation between material loss and the degree of stress. Among them, the stone-planting process performs the best, maintaining the lowest loss rate at all stages of quality loss, especially at the condition of 1000 rotational revolutions, and its loss rate is only 35.2%, which is 17.7 percentage points lower than that of the worst-performing glossy process (52.9%), which is a very significant advantage.

Further analysis of the loss growth trend can be found, indicating that the loss of the glossy process has the fastest growth rate, and with the increase in the number of rotational turns from 100 to 1000, the mass loss increased by 44.6%, while the stone-planting process only increased by 28.7%, which shows that the quality of the process not only reduces the value of the absolute loss but also effectively slows down the growth rate of the loss. From the practical application point of view, in the occasions that need to withstand large mechanical loads for a long time, the stone-planting or stone exposure process is obviously the best choice, although its initial cost may be higher, but in the long run it can significantly prolong the service life; for light loads or short-term use of the scenario, the lower cost of the rough surface or grooving process may be more cost-effective. It is suggested that subsequent research can combine the cost of the process, implementation difficulty, and other factors to carry out a more comprehensive evaluation of the economic benefits, to provide a better basis for decision making in engineering practice.

## 4. Conclusions

This paper analyzes the influencing factors on the slip resistance characteristics of high-flow cement concrete pavements through pendulum value and constructive depth, and its conclusions are as follows:

(1) It is concluded from the experimental analysis that dolerite, relative to basalt and limestone, blended into high-flow concrete will provide higher skid resistance and is an ideal type of stone-planting material.
(2) When dolerite is selected as the stone-planting material, the best anti-slip effect can be obtained when the stone spreading rate is 40%, the stone particle size grading is 13.2 mm, and the value of the stone pressed into the pavement with the depth of indentation is 0.8.
(3) The pendulum value of the pavement treated with stone planting increased by 40%, and the construction depth increased by 16.8 times relative to the bare pavement.
(4) The fort Kentucky flyaway test discovered that, under the same process conditions, the planted stone material being gabbro had a stone spreading area of 40%, stone size gradation of 13.2 mm, indentation depth ratio of 0.8, and the best wear-resistant cement concrete panels.
(5) Concrete stone-planting technology reduces the input of labor and machinery and improves the construction efficiency and construction process. At the same time, the application of stone-planting technology gives it excellent road performance such as anti-skid and wear resistance.

Due to the surface texture characteristics of the stone-planted cement concrete pavement, when the pendulum value is used to characterize the anti-slip performance of the pavement, the test data deviation is large and random, and it is impossible to accurately evaluate the anti-slip performance of the stone-planted cement concrete pavement. In this paper, the method of spreading gravel manually makes it difficult to ensure the uniformity of gravel spreading and the quality of gravel planting, and it is hoped that a more reasonable method can be found to ensure the uniformity of gravel planting in the future.

Stone-planted pavement construction has strict requirements for construction equipment (brushing machine pressure) and worker proficiency. Secondly, during long-term maintenance, loose stones may need to be filled periodically. Finally, a balance between skid resistance and driving comfort (noise, smoothness) is needed in future studies.

## Figures and Tables

**Table 1:** Test indicators and results of cement.

Projects			Test Results	Technical Requirement
Quasi-thickness water consumption (%)			28.1	-
Fineness (%)			1.3	≤30%
Stability (Ray’s Clamp Method)			0.49	≤5%
Condensation time (min)	Initial condensation time		150	≥45
	Final setting time		245	≤600
Strength (MPa)	Compressive strengths	7 d	34.1	≥17
		28 d	45.8	≥42.5
	Flexural strength	7 d	4.8	≥3.5
		28 d	8.5	≥6.5

**Table 2:** Physical properties of coarse aggregates.

Projects		Test Results	Technical Requirement
Apparent density (kg/m^3^)		2830	≥2500
Bulk density (kg/m^3^)	Loose	1118	-
	Terse	1580	≥1350
Clod content (%)		0.5	≤0.5
Mud content (%)		0.8	≤1
Needle flake content (%)		5.3	≤15
Saturated water absorption (%)		0.64	-

**Table 3:** River sand physical properties test results.

Projects	River Sand	Technical Requirement
Fineness Module	2.9	-
Apparent density (kg/m^3^)	2700	≥2500
Porosity (%)	35	-
Mud content (%)	0.2	-

**Table 4:** Naphthalene high-efficiency water reducing agent test report.

Projects	Test Results	Technical Requirement
Exterior	Yellow	Yellow
Water reduction rate	20	≥14%
PH value	8	6–9
Natrium sulfate	8.3	8–10
Net cement slurry flow rate	230 mm	≥140 mm
Mortar water reduction rate	19.1%	≥15%
Solid	92%	≥90%
Total alkali content	3.22%	≤5%

**Table 5:** Test indicators for diorite, basalt, and limestone aggregates.

Projects	Dolerite	Basalt	Limestone	Technical Requirement
Apparent density (kg/m^3^)	2986	2983	2730	≥2500
Water absorption (%)	0.6	1.10	0.68	≤2.0
Crushing value (%)	15.5	12.3	21.6	≤26
Content of elongated flat particles (%)	7.4	7.2	7.4	≤20
Los Angeles abrasion loss (%)	16.6	15.4	22.1	≤30
Buffing value (BPN)	45	45	42	≥42
Fine aggregate angularity (S)	38.6	39.7	32.8	≥30
Firmness (%)	2.8	3.1	2.2	≤5
Comprehensive index of anti-slip and abrasion resistance (t)	1.748	2.019	1.287	-

**Table 6:** Measurement results of stone spreading density.

Stone Size	Stone Types		
	Dolerite	Limestone	Basalt
9.5	13.15	12.18	13.32
13.2	17.99	15.97	16.17
9.5:13.2 = 1:1	15.57	14.08	14.75
9.5:13.2 = 2:1	14.76	13.44	14.27

## Data Availability

The original contributions presented in this study are included in this article. Further inquiries can be directed to the corresponding author.
